# Effect of container shape on freeze concentration of apple juice

**DOI:** 10.1371/journal.pone.0245606

**Published:** 2021-01-15

**Authors:** Tsuyoshi Yoda, Hiroshi Miyaki, Tomoaki Saito

**Affiliations:** Aomori Prefectural Industrial Technology Research Center, Hirosaki Industrial Research Institute, Hirosaki City, Aomori, Japan; University of Missouri Columbia, UNITED STATES

## Abstract

Concentrating fruit juices by freezing supports the maintenance of both nutrients and flavors. Development of the freezing concentration process has introduced equipment such as centrifuge or block freezing systems, which are suitable for large-scale commercial processing. However, the necessary characteristics of freeze concentration methods for juices include simplicity and low cost. This study examined the effects of different container shapes on the processes of freezing and melting. The shape of the container was found to be more important than the melting temperature, across a relatively large scale. Furthermore, the nutrient procyanidin B2 and saccharides were concentrated. The methods concentrated juice components under low cost conditions without complex equipment. This research thus not only offers benefits for commercial juice preparation but also provides new insight into effects of shape differences in concentration technologies.

## Introduction

Juice concentration by freezing is a widely studied process [1]. Its primary advantage is superior retention of flavors compared to concentration by heating [2]. Previous research reported that humans perceive reconstituted frozen concentrated juice as the same as fresh juice [2]. Freeze concentration of grape juice for making wine has been introduced by one of the biggest beverage production companies in Japan [3]. Freeze concentration of orange juice using freezing units has also been investigated [4]. Recently, combing both centrifuge and block freeze concentration processes yielded high-quality concentrated juice from blueberries and pineapples [5]. Because such a procedure is efficient in concentrating juices, manufacturers will introduce related methods and equipment.

Simple processes without a need for large machines or instrumentation would enable small agriculture companies to use these techniques. Therefore, this study sought to develop a simple concentration system and to evaluate the quality for the product using under the several simple conditions. Simple concentration methods have been reported in the past. One of these is the ice cube tray method [6, 7]. Freezing and thawing using ice cubic trays can concentrate a 1% concentration solution of sodium chloride, glucose, fructose, lactose, and sucrose by 4–5 fold [6]. When such saccharides were concentrated with sodium chloride, increasing the concentration of chloride, there was a decrease in concentration because of freezing point depression [7].

A Japanese patent reported similar results, with about six-fold concentration, by freezing an extract of crab that contained saccharides at Brix 2%–3% [8]. The process used an instrument similar to a separatory funnel.

These reports provided profiles of freeze concentration using the ice cube tray or separatory funnel [6–8]. However, they used beverage materials with low concentrations of saccharides, at 2%–3% maximum [8]. This study attempted to apply such previously reported methods to apple juice, which contains more than 10% saccharides.

This study also aimed to expand the process to a relatively large scale for freeze concentrating juice in amounts of 13 L and 18 L. For many types of fruit juice, the effects on flavor quality after concentration by various methods has been investigated. Some studies evaluated components using gas chromatography–mass spectrometry (GC–MS) [1, 9–11]. Human tasting is also used to test apple juice [12], and the concentrated apple juice is often used to study the juice qualities [13, 14]. Apple polyphenol is a valued nutrient that decreases the level of low-density lipoprotein cholesterol [15]. One apple polyphenol ingredient focused on is procyanidin B2 [16]. Its presence can be measured using high-performance liquid chromatography (HPLC) [16, 17]. Therefore, this study measured the procyanidin B2 levels in concentrated products, applying the process at a relatively large scale.

This research aimed to derive simple and effective concentration methods and to document the profile of freeze-concentrated juice. It also investigated a relatively large-scale process with concentration effects on procyanidin B2. As far as we know, this is the first report to compare effects of the freezing tray shape and the quality of the resulting concentrated juice. It may have uses in freeze concentration not only of apple juice but of many other kinds of beverages.

Therefore, the purpose of the present study is to find simple methods using different types of containers and to develop relatively large-scale processes. To resolve points or more simply to know the properties change during melting temperature and shape of container. The present study aimed to understand the characteristics of condensed juice in several conditions of temperature and two types of shapes and to develop a relative large-scale process for investigating the contents of both sugar and procyanidin B2 as indicators.

## Materials and methods

### Samples and reagents

Pure apple juice (1 L bottles) was purchased from Japan Agricultural Cooperatives (Aomori, Japan) for small-scale examination. Pure apple juice for commercial use (18 L can) was obtained from Aomori Prefecture Juice LTD (Aomori, Japan) for relatively large-scale application. Methanol, acetone, acetonitrile, acetic acid and procyanidin B2 were obtained from Fuji Film Wako Pure Chemical, Japan. Ultrapure water from a Millipore Milli-Q purification system (Millipore, Bedford, MA, USA) was used.

### Freeze concentration

**Fig 1:**
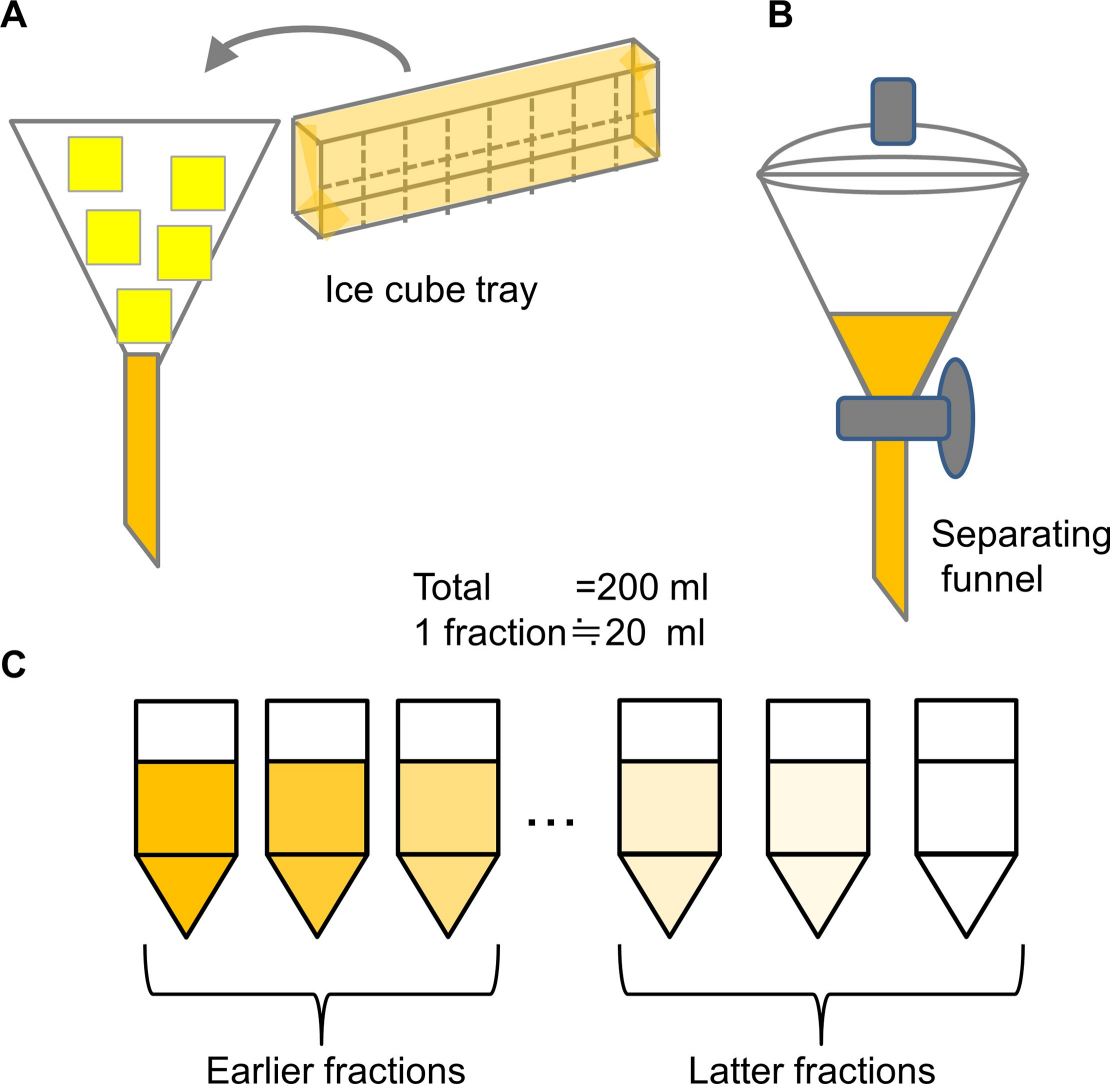
Experimental procedure for freezing and thawing of juice. Using an ice cube tray with funnel (A), separatory funnel (B), and showing fractions (C).

Using a mass-marketed freezer (Hoshizaki Corporation, Aichi, Japan), 200 ml of apple juice was frozen with an ice cube tray [6, 7] or a separatory funnel [8] as shown in Scheme 1 to allow small-scale examination. The juice concentrates obtained were allowed to melt in a room set to a temperature of 5°C, 10°C, or 20°C. The rooms were maintained at these temperatures in our institute for materials storage. The experimental procedure is shown in Fig 1. These experiments were repeated at least three times.

### Larger scale examination

This experiment used 18 L apple juice provided for commercial use. The 18 L of apple juice was placed in a 20-L water tank and 13 L of apple juice was placed in a 15-L tank to assess freeze expansion. These were then stored in a mass-marketed freezer at −18°C over a weekend. These frozen tanks were melted in a 10°C room for small-scale examination. A large-scale experiment was also carried out using either a 20-L washable tank (made by Iwatani Materials LTD, Tokyo, Japan) or a 15-L plastic tank (folding water tank, PW-15, made in China). The melting process is shown in S1 Video. Approximately 1.8 L and 1.3 L of each fraction was collected in the two versions, respectively. Each fraction was collected in a beaker and stored in a 1.8-L bottle. To provide a concentrated juice (labeled “condensed”), the first through third fractions (in total for the two versions, six fractions) were combined. The fourth through 10th fractions were combined as the “rest” of the material (in total for the two versions, 14 fractions).

### Measurement of sugar content

Sugar levels as Brix values were measured using a PR-201a digital refractometer (Atago, Tokyo, Japan) that had been calibrated to a value of 0 with ultrapure water.

### Measurement of procyanidin B2

A 25-mL measurement sample was prepared by mixing 5 mL of apple juice, 17.5 mL of acetone, and pure water. The filtered sample solution was used for procyanidin B2 measurement by HPLC, Elite LaChrom system, Hitachi, Japan) with Inertsil WP 300 Diol (GL Sciences Inc., Japan) using an Elite LaChrom L-2130 quaternary pump and an Elite LaChrom L-2420 detector [16–20] at 35°C. Mixtures of acetonitrile and acetic acid (mobile phase A, acetonitrile: acetic acid = 98:2) and methanol, water and acetic acid (mobile phase B, methanol: water: acetic acid = 95:3:2) were used as the mobile phases. Elution was performed using a linear gradient of 0–7% B for 0–3.0 min, followed by a linear gradient of 7–30% B for 57.0 min. Subsequently, mobile phase B was increased from 30% to 100% over 60.0–70.0 min. The mobile phase was subsequently returned to initial conditions (0% B) to re-equilibrate for 10.0 min. The injection volume was 5 μL using auto sampler, the flow rate was set at 1.0 mL/min, and fluorescence detection of procyanidins B2 was performed with excitation and emission wavelengths of 230nm and 321 nm, respectively. It was confirmed that the retention time was the same for procyanidin B2 for standard and sample for quantification in same measurements. The calibration curve fittings were carried out on a personal computer using the Excel program (*R*^2^ > 0.99) with pure procyanidin B2 in a methanol/water (1/1 volume) solution.

### Statistical analysis

Data of Brix values are expressed as mean and standard error; the data were analyzed using Microsoft Excel. Two-way ANOVA in Excel was used to test the differences for comparison of frozen and melted results in accordance with temperature and container shape.

## Results and discussion

### The effect of melting temperature and container shape

**Fig 2:**
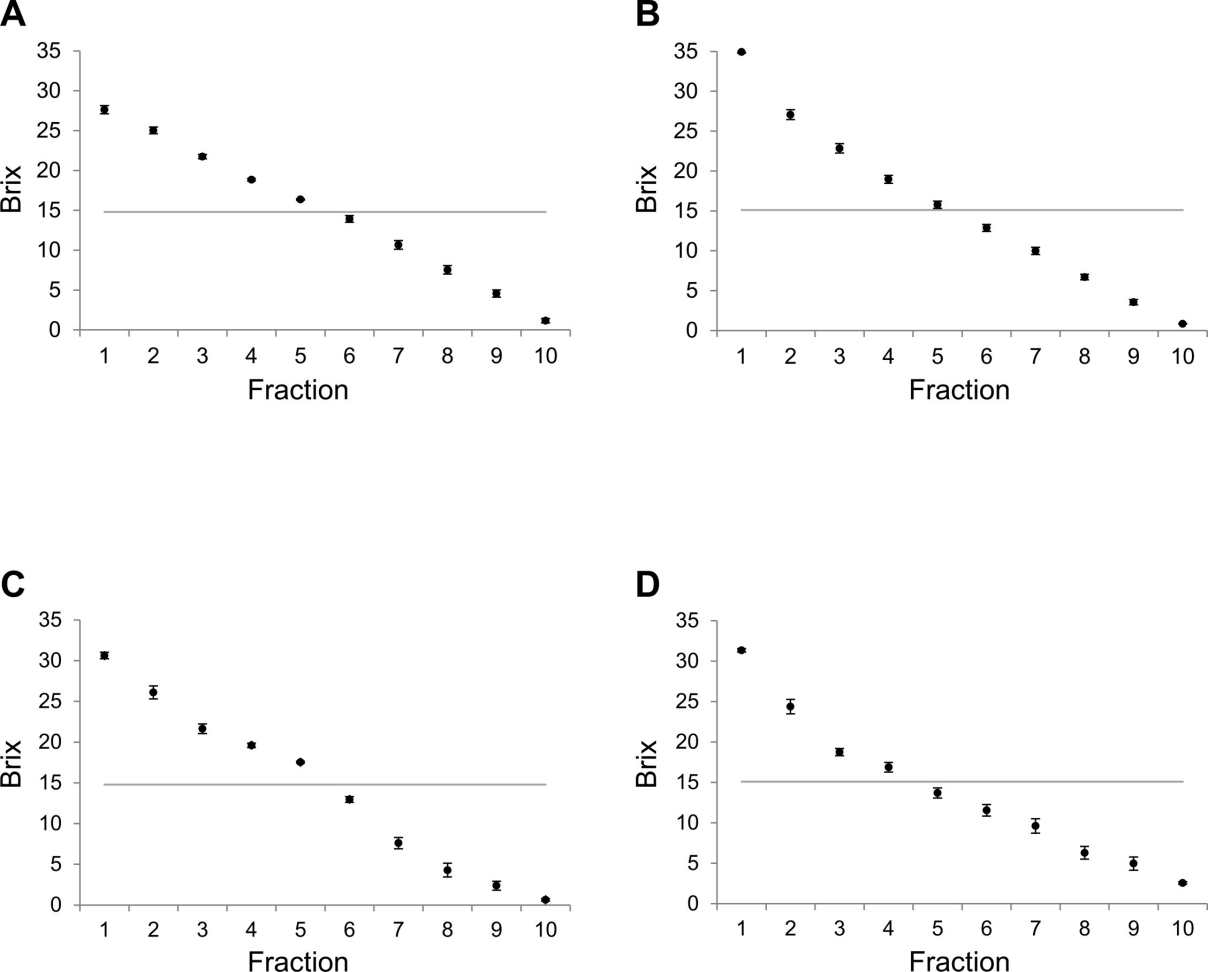
Brix values (dots) after freezing and thawing under several conditions. Using an ice cube tray with funnel (A and C), a separatory funnel (B and D), in A and B at 10°C and in C and D at 20°C. Each gray line indicates the Brix values of fresh juice. The experiments were conducted at least three times. The Brix values were expressed as mean and standard error.

The effects for freeze concentration of different shapes and melting conditions with surrounding temperature are shown in Fig 2. All experiments were repeated at least three times, and the standard error each time was considerably small. Therefore, the Brix values were considered reliable. The Brix value of original juice was 15°. To evaluate condensed juice, the mean Brix values of each fraction were calculated and are shown in Table 1. At 10°C, the mean values for the separatory funnel method of the first through ninth fractions are larger than the values obtained using by the ice cube tray method, and the same applied at 20°C (Table 1). Therefore, freeze concentration using a separatory funnel is a more effective concentration method than the ice cube tray under both temperature conditions (20°C and 10°C). At 20°C, some sticky material remained in both the ice cube tray and the separatory funnel, which may cause the total Brix to be less than 15°. A solution of more than twice the concentration, as shown by the Brix value, could be obtained in first 10% fraction. Yee et al. reported that for thawing carried out at 10°C or room temperature, the results were similar [6]. Our results are also similar under two conditions (20°C and 10°C), although our previous study used sodium chloride and saccharides.

**Table 1:** Fraction(s) and their mean Brix values.

Fraction(s)	Mean Brix					
	Melting temperature 20°C		Melting temperature 10°C		Melting temperature 5°C	
	Ice cube tray	Separatory funnel	Ice cube tray	Separatory funnel	Ice cube tray	Separatory funnel
1	31	31	28	35	28	33
1 + 2	28	28	26	31	25	30
1 + 2 + 3	25	26	25	28	23	27
1 + 2 + 3 + 4	23	24	23	26	21	25
1 + 2 + 3 + 4 + 5	21	23	22	24	20	23
1 + 2 + 3 + 4 + 5 + 6	19	21	21	22	18	21
1 + 2 + 3 + 4 + 5 + 6 + 7	18	19	19	20	16	20
1 + 2 + 3 + 4 + 5 + 6 + 7 + 8	17	18	18	19	15	18
1 + 2 + 3 + 4 + 5 + 6 + 7 + 8 + 9	15	16	16	17	14	16
1 + 2 + 3 + 4 + 5 + 6 + 7 + 8 + 9 + 10	14	14	15	15	12	15

The mean Brix value of each fraction was calculated to evaluate the efficiency of tray shape at 20°C, 10°C, and 5°C.

**Fig 3:**
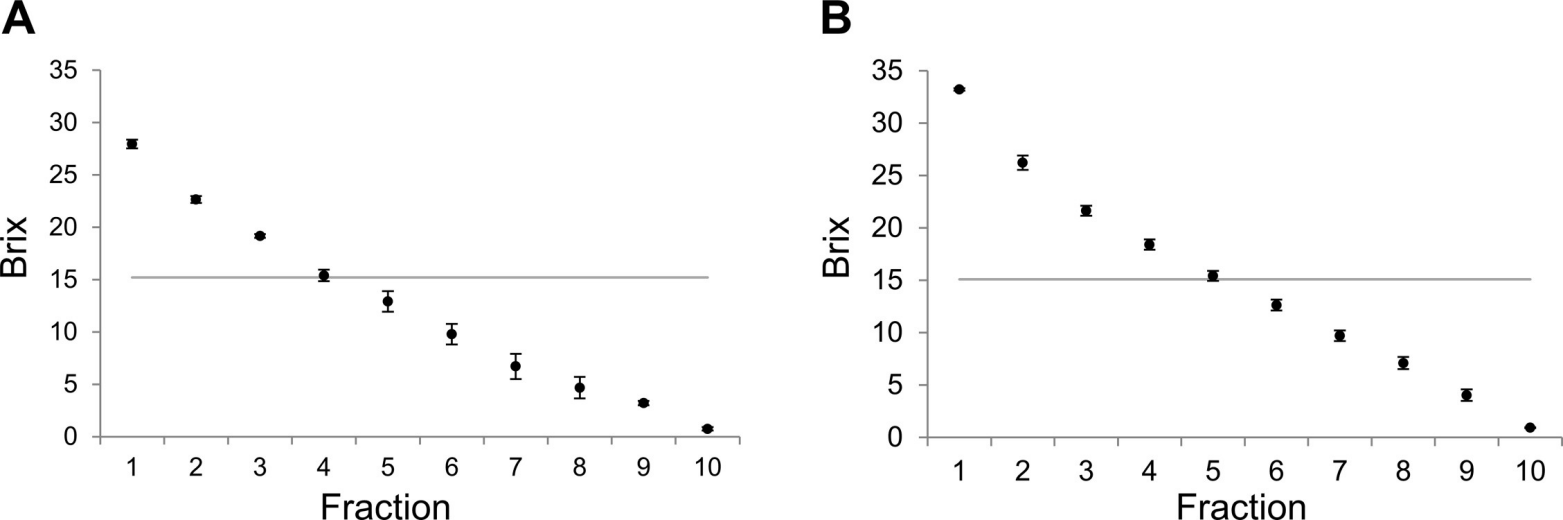
Concentration profile for Brix values after freezing and thawing at 5°C. Using an ice cube tray with funnel (A) and separatory funnel (B) are shown. Each gray line indicates the Brix value of fresh juice. The experiments were performed at least three times. The Brix values were expressed as mean and standard error.

Next, effects of a colder condition were investigated, for when the temperature reaches about 5°C. The effects of different shapes and melting conditions for freeze concentration at 5°C are shown in Fig 3. As for the processes at 20°C and 10°C, freeze concentration using a separatory funnel is more effective in concentration than using an ice cube tray at 5°C with Brix values of first through fifth fractions with the funnel (Fig 3B) being higher than for the ice cube tray (Fig 3A). Both experiments were repeated at least three times, and the standard error each time was considerably small. Therefore, the Brix values at 5°C, 10°C, and 20°C were considered reliable. For the condensed juice, mean Brix for each fraction were calculated and are provided in Table 1. The mean values for the first to first to 10th fractions using the separatory funnel are larger than the values obtained using the ice cube tray. When there is a sticky remainder in the ice tray, this may cause a total Brix value less than 15°.

Here, we compare these results. At 20°C, the Brix values are significantly different between ice cube tray with separatory funnel, over six fractions (Table 1). Likewise, the difference between ice cube tray with separatory funnel, over three fractions are significant at both 20°C and 5°C conditions (two-way ANOVA, p < 0.05). In ice cube tray, the values are not significantly different between 20°C and 10°C even up to 10 fractions (two-way ANOVA, p > 0.05). Significant difference was also seen at 10°C–5°C, over five fractions compared with 20°C–5°C, over three fractions are significantly different (two-way ANOVA, p < 0.05). While using a separatory funnel, the values are significantly different between 20°C and 10°C three fractions (two-way ANOVA, p < 0.05). Similarly, there was significant difference at 10°C–5°C, over four fractions compared with 20°C to 5°C, over three fractions are significantly different (two-way ANOVA, p < 0.05). Even difference melting temperature 20°C–5°C, small amount of fractions difference in same container shape, container shape difference make significant difference relatively smaller fraction in same temperature.

These results demonstrate that the shape of the container is more important than the melting temperature in producing concentrated apple juice by freeze concentration and melting. This is the first report to investigate differences in the shape of the container for freeze concentration of apple juice. How the shape affects freeze concentration when melting solid ice to liquid juice is not clear. Although a similar phenomenon was reported in a patent when using a setup like a separatory funnel for freeze concentration with low concentrations of saccharides in solution [8], the mechanism remains unclear.

### Large-scale application

This study also aimed to apply these phenomena to industrial use by scaling up to a relatively large scale, around 20 L. A large separatory funnel could not be obtained, so two types of washable water tank were prepared.

**Fig 4:**
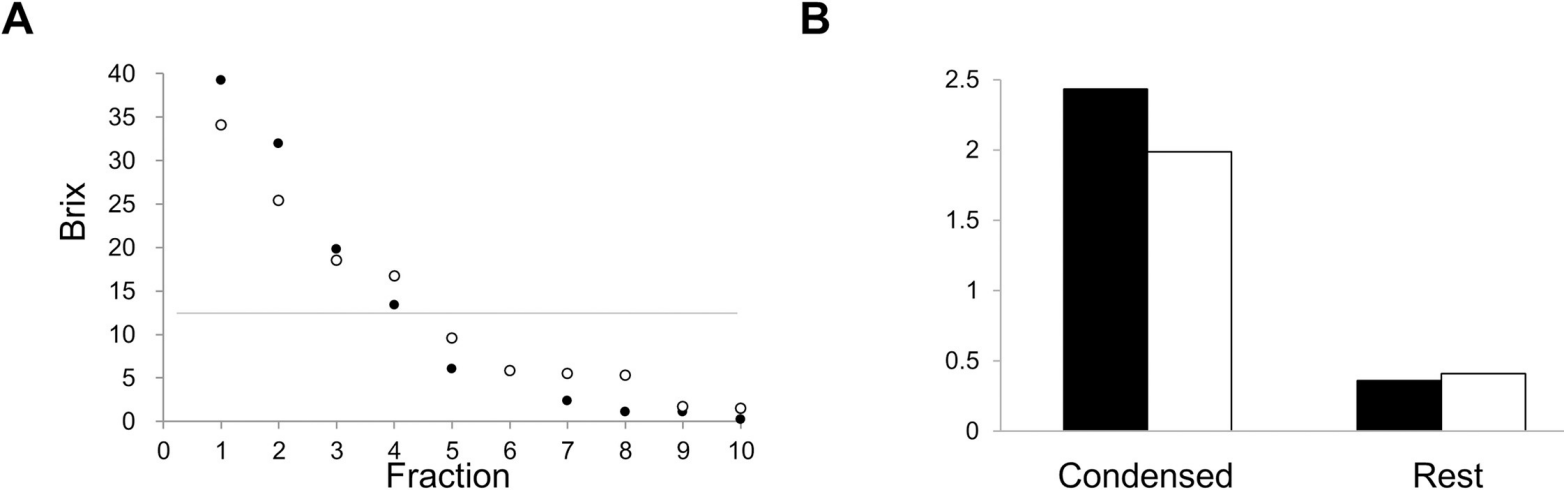
Concentration profiles for Brix values and procyanidin B2 after freezing with a large-scale application. (A) The two scales, 18 L (black) and 13 L (white), and their Brix value at each fraction. (B) are shown. The Brix values and procyanidin B2 levels for original juice. The first through third factions are mixed as “Condensed,” and the “Rest” is the fourth through 10th fractions.

In this part of the study, commercial apple juice was used with a Brix value of 13°. The frozen apple juice in water tanks was melted in a 10°C room (see the description in the results of the small-scale examination). They were investigated under two conditions: 13 L and 18 L of apple juice. Each fraction in each condition was collected, and their Brix values are shown in Fig 4A. The larger amount of apple juice, 18 L, was well concentrated in the early fractions. The Brix value of the first fraction showed more highly concentration than for the small-scale concentration process. Using 13 L the apple juice was well concentrated also, and more so than with the ice cube tray process. Accordingly, both water tanks are as well suited to concentrating the juice as the process that used the separatory funnel. In total, 20 fractions were selected as either concentrated juice (labeled “condensed”) or the “rest.” The first to third fractions (in total, six fractions) were mixed as frozen, and the “rest” included the fourth to 10th fractions (in total, 14 fractions). Their Brix values were 30 and 4.5°, respectively. Then it was investigated to confirm concentration for the focused nutrient procyanidin B2 in freezing concentrations.

### Concentration of procyanidin B2

The concentration of the nutrient factor procyanidin B2 was measured in the original juice, and in the freeze concentrated, sorting the values into condensed and the rest, compared to the value in the original juice (set to 1 on the scale) (Fig 4B). The actual value was a content of 8.3 mg procyanidin B2 per 100 g in the original juice. This value agreed with previous reports [16]. The other actual values of procyanidin B2 were 17 mg per 100 g and 3.0 mg per 100 g for the “condensed” and the “rest,” respectively. Procyanidin B2 was concentrated along with sugar. These concentrated levels were about twice as large as in the original juice (Fig 4B). This indicates better concentration effects, using these freeze and melt methods, because the concentration affected not only saccharides but also the nutrient procyanidin B2 at a relatively large scale. These effects provide advantages in preparing resources for foods and beverages. Another merit of this method is the low cost, because the water tanks are inexpensive and can be recycled for many uses.

One of the advantages of this methodology indicated by our results is as follows: forming ice block as a single block is better than generating multiple ice blocks separately. This is attributable to the fact that it is possible to ensure that the ice blocks are more densely concentrated when formed as a single block one than as multiple separate blocks. Furthermore, this concept could have relatively large scale applications, such as the concentration of procyanidin B2 levels in myriad products. This methodology can be considered useful when preparing concentrated solutions such as juices. Such concentrated juices are used as ingredients of alcoholic beverages [1, 3] and seasonings, such as sauce [8], among other applications; therefore, our results will be useful in the food and beverage manufacturing industry.

### Comparison with other fruit juice concentration methods

The most consumed fruits used as juice in the world are apple and orange [21]. The third one is grape. The ranking is same in Japan too. Freezing and melting to condense may be applicable not only to apple but also to other fruits such as orange and grape. Several concentration methods are reported for such fruits [1–5]. Condensed juices made from such fruits are used as materials to make alcoholic beverages. The knowledge and methods in the present study would be useful to make condensed juice in an effectual and economical manner.

## Conclusions

The effects of container shape difference are more significant than those of the melting temperature. The phenomenon held at a relatively large scale. Further, the nutrient procyanidin B2, as well as the saccharides, could be concentrated in this process. These methods provide concentration of apple juice components at low cost without complex machinery. The results not only should benefit various juice manufacturers but also provide novel insight into effects of shape difference in concentration technologies.

## Supporting information

S1 Video (MP4)

## References

[1] Miyawaki O, Gunathilake M, Omote C, Koyanagi T, Sasaki T, Take H, et al Progressive freeze-concentration of apple juice and its application to produce a new type apple wine. Journal of Food Engineering, 2016, 171, 153–158. 10.1016/j.jfoodeng.2015.10.022.

[2] Sánchez J, Ruiz Y, Auleda JM, Hernández E, Raventós M. Review. Freeze Concentration in the Fruit Juices Industry. Food Science and Technology International, 2009, 15(4), 303–315. 10.1177/1082013209344267.

[3] Suntory. Freezing and concentration method of Koshu grape juice. Retrieved from https://www.suntory.co.jp/wine/nihon/blog/15112016.html.(accesed September 22, 2020)

[4] Sánchez J, Ruiz Y, Raventós M, Auleda JM, Hernández E. Progressive freeze concentration of orange juice in a pilot plant falling film. Innovative Food Science and Emerging Technologies, 2010, 11(4), 644–651. 10.1016/j.ifset.2010.06.006.

[5] Petzold G., Moreno J., Lastra P., Rojas K, Orellana P. Block freeze concentration assisted by centrifugation applied to blueberry and pineapple juices. Innovative Food Science and Emerging Technologies, 2015, 30, 192–197. 10.1016/j.ifset.2015.03.007.

[6] Yee PL, Wakisaka M, Shirai Y, Hassan MA. Effects of Single Food Components on Freeze Concentration by Freezing and Thawing Technique. Japan Journal of Food Engineering, 2003, 4(3), 77–83.

[7] Yee PL, Wakisaka M, Shirai Y, Hassan MA. Effect of Sodium Chloride on Freeze Concentration of Food Components by Freezing and Thawing Technique. Japan Journal of Food Engineering, 2004, 5(2), 97–102.

[8] Japan Patent. 2008. P4081514 (2008.2.22).

[9] Ramos FA, Delgado JL, Bautista E, Morales AL, Duque C. Changes in volatiles with the application of progressive freeze-concentration to Andes berry (Rubus glaucus Benth). Journal of Food Engineering, 2005, 69(3), 291–297. 10.1016/j.jfoodeng.2004.07.022.

[10] Gunathilake M, Shimura K, Dozen M, Miyawaki O. Flavor Retention in Progressive Freeze-Concentration of Coffee Extract and Pear (La France) Juice Flavor Condensate. Food Science and Technology Research, 2014, 20(3), 547–554. 10.3136/fstr.20.547.

[11] Tabata S, Iida K, Suzuki J, Kimura K, Ibe A, Saito K. A Quantification and Confirmation Method of Patulin in Apple Juice by GC/MS, Food Hygiene and Safety Science,2004, 45(5), 245–249. 10.3358/shokueishi.45.245 15678938

[12] Yoshimoto A, Yahada A, Yamamoto K, Sasaki K, Funakosi-Yoshida A, Ohta H. The Quality of Concentrated Orange and Apple Juice with Relation to the Content of Saccharide and Organic Acid (in Japanese). Nakamuragakuendaigaku, Nakamuragakuendaigakutankidaigakubukenkyukiyo, 2013, 45, 183–193. https://nakamurau.repo.nii.ac.jp/?action=repository_action_common_download&item_id=187&item_no=1&attribute_id=22&file_no=1.

[13] Shirasawa S, Shiota M, Arakawa H, Shigematsu Y, Yokomizo K, Shionoya N, et al Quantitative Determination of Trans-Fatty Acids in Oils and Fats by Capillary Gas Chromatography: Results of a JOCS Collaborative Study. Journal of Oleo Science, 2007 56(8), 405–415. 10.5650/jos.56.405 17898507

[14] Jeleń HH, Majcher M, Dziadas, M. Microextraction techniques in the analysis of food flavor compounds: A review. Analytica Chimica Acta, 2012, 738, 13–26. 10.1016/j.aca.2012.06.006 22790695

[15] Nagasako-Akazome Y, Kanda T, Ohtake Y, Shimasaki H, Kobayashi T. Apple Polyphenols Influence Cholesterol Metabolism in Healthy Subjects with Relatively High Body Mass Index. Journal of Oleo Science, 2007, 56(8), 417–428. 10.5650/jos.56.417 17898508

[16] Shoji T, Methods of detection for procyanidins on apples (English title was translated by the authors, written in Japanese) Retrieved from http://www.google.co.jp/url?sa=t&rct=j&q=&esrc=s&source=web&cd=&ved=2ahUKEwj3-qrVm4ntAhUJa94KHYsjCkEQFjAAegQIAxAC&url=http%3A%2F%2Ffmric.or.jp%2Fffd%2Fffmanual%2Fmanual40107.pdf&usg=AOvVaw38wtp4L_mmnBh4UH1Vre88

[17] Yamazaki S, Nakada M, Osawa K. Study on Functional Ingredients Contained in Apples and Leeks Produced in Nagano Prefecture.2017 Retrieved from https://www.gitc.pref.nagano.lg.jp/reports/pdf/H29/H29F19.pdf.

[18] Obara M, Matsumoto S, Ono Y, Ozaki Y, Shoji T. Procyanidin concentrations and H-ORAC of Apples Cultivated in Japan, Food Science and Technology Research, 2016, 22(4), 563–568. 10.3136/fstr.22.563

[19] Shoji T, Masumoto S, Moriichi N, Kanda T, Ohtake Y. Apple (Malus pumila) procyanidins fractionated according to the degree of polymerization using normal-phase chromatography and characterized by HPLC-ESI/MS and MALDI-TOF/MS. J Chromatogr A, 2006, 1102, 206–213. 10.1016/j.chroma.2005.10.065 16313915

[20] Hammerstone JF, Lazarus SA, Mitchell AE, Rucker R, Schmitz HH. Identification of Procyanidins in Cocoa (Theobroma cacao) and Chocolate Using High Performance Liquid Chromatography/Mass Spectrometry. Journal of Agricultural and Food Chemistry, 1999, 47(2), 490−496. 10.1021/jf980760h 10563922

[21] Distribution status of fruit juice (written in Japanese, the title translated by the authors) Japan Consumer Affairs Agency 2010 July Retrieved from https://www.cao.go.jp/consumer/doc/100721_shiryou1-4.pdf

